# Management of X-Linked Hypophosphatemia During Pregnancy: Case Report and Literature Review

**DOI:** 10.1007/s00223-026-01495-w

**Published:** 2026-02-12

**Authors:** Gaetano Paride Arcidiacono, Mor Peleg Falb, Marco Onofrio Torres, Valentina Camozzi, Martin Diogo, Francesca Guidolin, Alberta Cecchinato, Elena Campello, Paolo Simioni, Sandro Giannini, Stefania Sella

**Affiliations:** 1 Department of Medicine, University of Padova, Via Giustiniani 2, 35128 Padua, Italy; 2 Clinica Medica 1, Department of Medicine, Azienda Ospedale-Università Padova, Padua, Italy; 3 Endocrinology Unit, Department of Medicine, Azienda Ospedale-Università Padova, Padua, Italy; 4 Metabolic Bone Disease Unit, Azienda Ospedale-Università Padova, Padua, Italy

**Keywords:** X-linked hypophosphatemia, Pregnancy, Phosphate, Rickets

## Abstract

Hypophosphatemia during pregnancy poses unique clinical challenges due to physiological changes in mineral metabolism, with potential impacts on maternal and fetal health, but limited evidence to guide treatment. We describe the case of a woman with X-linked hypophosphatemia, previously treated with burosumab for lower limb pseudofractures. Due to her desire for pregnancy and the absence of safety data on burosumab during gestation, the drug was discontinued and treatment with phosphate supplements and calcitriol was initiated. Following assisted reproduction with preimplantation genetic diagnosis, the patient successfully carried the pregnancy to term and delivered a healthy newborn. Monthly biochemical monitoring allowed for safe adjustment of therapy, maintaining serum phosphate levels within or near the normal range without maternal or fetal complications. We also review the available literature on hypophosphatemia in pregnancy, emphasizing the need for individualized management and close monitoring.

## Significance Statement

The management of X-linked hypophosphatemia (XLH) during pregnancy poses significant clinical challenges due to physiological changes in mineral metabolism and the absence of evidence-based guidelines. This case report describes a successful pregnancy in a woman with XLH treated with conventional phosphate and calcitriol supplementation, closely monitored throughout gestation. Maternal and neonatal outcomes were favourable, with no reported complications. By combining clinical experience with a targeted literature review, this report helps fill a critical knowledge gap and offers clinicians a concrete example of how to safely manage hypophosphatemia during pregnancy, supporting maternal skeletal health and optimal fetal development.

## Introduction

Chronic hypophosphatemia is a condition that may lead to impaired bone mineralization, resulting in rickets and/or osteomalacia, with significant skeletal complications such as bone pain, skeletal deformities, and pseudofractures or fractures [1]. Among the causes of chronic hypophosphatemia, X-linked hypophosphatemia (XLH) is the most common inherited form. XLH is caused by *PHEX* gene mutations that lead to elevated serum fibroblast growth factor 23 (FGF23) levels, which in turn cause hypophosphatemia both through renal phosphate wasting due to reduced tubular phosphate reabsorption and decreased intestinal phosphate absorption resulting from reduced synthesis of 1,25-dihydroxyvitamin D (1,25[OH]_2_D) [2].

Conventional treatment for XLH is based on oral phosphate supplements combined with active vitamin D analogs, such as calcitriol or alfacalcidol, which are often associated with side effects that can limit patient compliance and compromise long-term outcomes [3]. However, burosumab, a monoclonal antibody against FGF23, has transformed the treatment of XLH by improving serum phosphate levels, promoting bone healing, reducing pain, and improving quality of life [4]. In 2023, the Italian Medicines Agency (AIFA) approved reimbursement of burosumab for adults with XLH who have active fractures or pseudofractures despite conventional treatment [5], and its effectiveness has also been confirmed in real-life clinical settings on clinical and laboratory outcomes [6].

However, burosumab has not been studied in pregnancy and, due to the absence of safety data, its use during gestation is currently not recommended [7]. Therefore, the management of hypophosphatemia during pregnancy remains challenging. Physiological changes in phosphate and calcium metabolism, potential maternal and fetal complications, and the lack of data on the safety of treatments during pregnancy contribute to this significant clinical uncertainty. Reports on pregnancy outcomes in women with XLH, but also in those with other hypophosphatemic disorders, are scarce and mainly limited to isolated case reports. We report the case of a woman with XLH complicated by pseudofractures, initially treated with burosumab, which was discontinued due to her desire for pregnancy. After transitioning to conventional therapy, she successfully carried the pregnancy to term and delivered a healthy newborn. We also provide a review of the current literature on the management of hypophosphatemia during pregnancy.

## Case Presentation

**Fig. 1:**
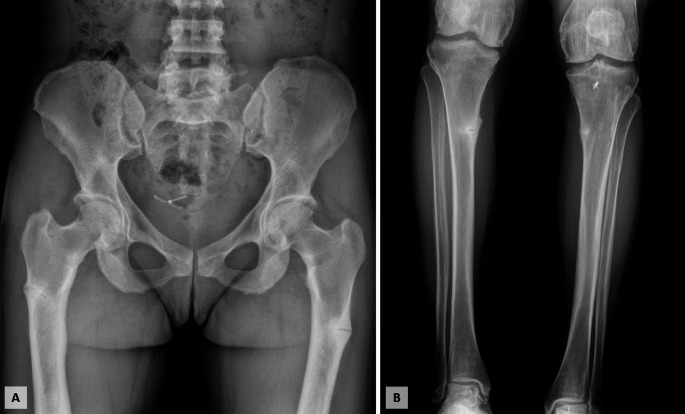
X-rays of the lower limbs showing multiple pseudofractures involving the femurs (**A**) and tibias (**B**)

We present the case of a 42-year-old woman with a diagnosis of XLH referred to our Center in 2023. The diagnosis was established at the age of 2 years at another institution, based on findings of low serum phosphate, poor growth, dental abnormalities, and lower limb deformities, which required multiple corrective orthopaedic surgeries and several dental interventions. In 2011, at the age of 28 years old, the patient underwent genetic testing that identified the presence of a heterozygous pathogenic PHEX variant, c.1645C > T (p.R549X). Her family history was negative for individuals with clinical features or a history suggestive of rickets, and both parents tested negative for PHEX mutations. Aside from XLH-related manifestations, her past medical history was unremarkable. At the time of our evaluation, she was receiving only phosphate supplements and calcitriol, a treatment that had been initiated during early childhood and has been continued since then. On physical examination, she was 150 cm tall and weighed 55 kg, with a body mass index (BMI) of 24.4 kg/m^2^; she exhibited bowed lower limbs and genu varum. Laboratory tests at the time of the patient’s initial evaluation at our Center revealed low serum phosphate levels, elevated urinary phosphate excretion as measured by the tubular maximum reabsorption of phosphate adjusted for glomerular filtration rate (TmP/GFR) [8], increased parathyroid hormone (PTH) levels, and inappropriately normal FGF23 levels for the degree of hypophosphatemia (Table 1). A dual-energy X-ray absorptiometry (DXA) scan showed Z-scores comparable to those of age- and sex-matched controls (lumbar spine + 0.8, total hip − 1.1, femoral neck − 0.3). The patient reported diffuse bone pain, stiffness, and joint limitation, which prompted radiographic evaluation and revealed multiple pseudofractures involving the lower limbs (Fig. 1).

**Table 1:** Laboratory tests at initial evaluation at our center

Laboratory test	Patient	Reference range
Serum calcium (mg/dL)	9.1	8.8–10.6
Serum phosphate (mg/dL)	**1.7**	2.5–4.5
Urinary calcium (mg/24 h)	248	100–300
Urinary phosphate (mg/24 h)	1153	400–1300
TmP/GFR (mg/dL)	**1.7**	2.2–3.6
Serum creatinine (mg/dL)	0.62	0.51–0.95
Creatinine clearance (mL/min)	120	> 90
25[OH]D (nmol/L)	73	70–125
1,25[OH]_2_D (pmol/L)	86.7	47.8–190.3
PTH (ng/L)	**43.9**	6.5–36.8
ALP (U/L)	80	30–120
CTX (pg/mL)	226	121–747
P1NP (µg/L)	52.5	28.0–128.0
Intact FGF23 (pg/mL)	50.2	23.2–95.4
C-Terminal FGF23 (pmol/L)	0.10	0.00–0.80

Values outside the reference range are highlighted in bold. 1,25[OH]_2_D = 1,25-dihydroxyvitamin D; 25[OH]D = 25-hydroxyvitamin D; ALP = alkaline phosphatase; CTX = C-terminal telopeptide of type I collagen; FGF23 = fibroblast growth factor 23; P1NP = N-terminal propeptide of type I procollagen; PTH = parathyroid hormone; TmP/GFR = tubular maximum for phosphate reabsorption adjusted for glomerular filtration rate

For this reason, in January 2024, after discontinuation of conventional therapy, treatment with burosumab was initiated at a dose of 60 mg subcutaneously every four weeks (equivalent to 1.0 mg/kg of body weight, rounded to the nearest 10 mg, every four weeks), in combination with cholecalciferol supplementation (7500 IU per week). Following the initiation of burosumab therapy, serum phosphate levels increased from 1.7 to 3.1 mg/dL 14 days after the injection, and were 2.7 mg/dL at day 28 (just before the next dose). This was accompanied by an improvement in TmP/GFR (2.4 mg/dL) at day 28, with no significant effect on renal function, serum and urinary calcium, and normalization of PTH levels. After eight months, serum phosphate levels remained elevated (3.1 mg/dL at the midpoint of the burosumab dosing interval), and the patient reported reductions in pain (in both frequency and intensity), improved joint stiffness, improved mobility, and a greater ability to perform activities of daily living, resulting in an overall improvement in quality of life, as evaluated by the Western Ontario and McMaster Universities Osteoarthritis Index (WOMAC) total score (from 48 to 36), the Brief Pain Inventory – Short Form (BPI-SF) worst pain score (from 9 to 5), and the Six-Minute Walk Test (6MWT) distance (from 420 to 442 m).

**Fig. 2:**
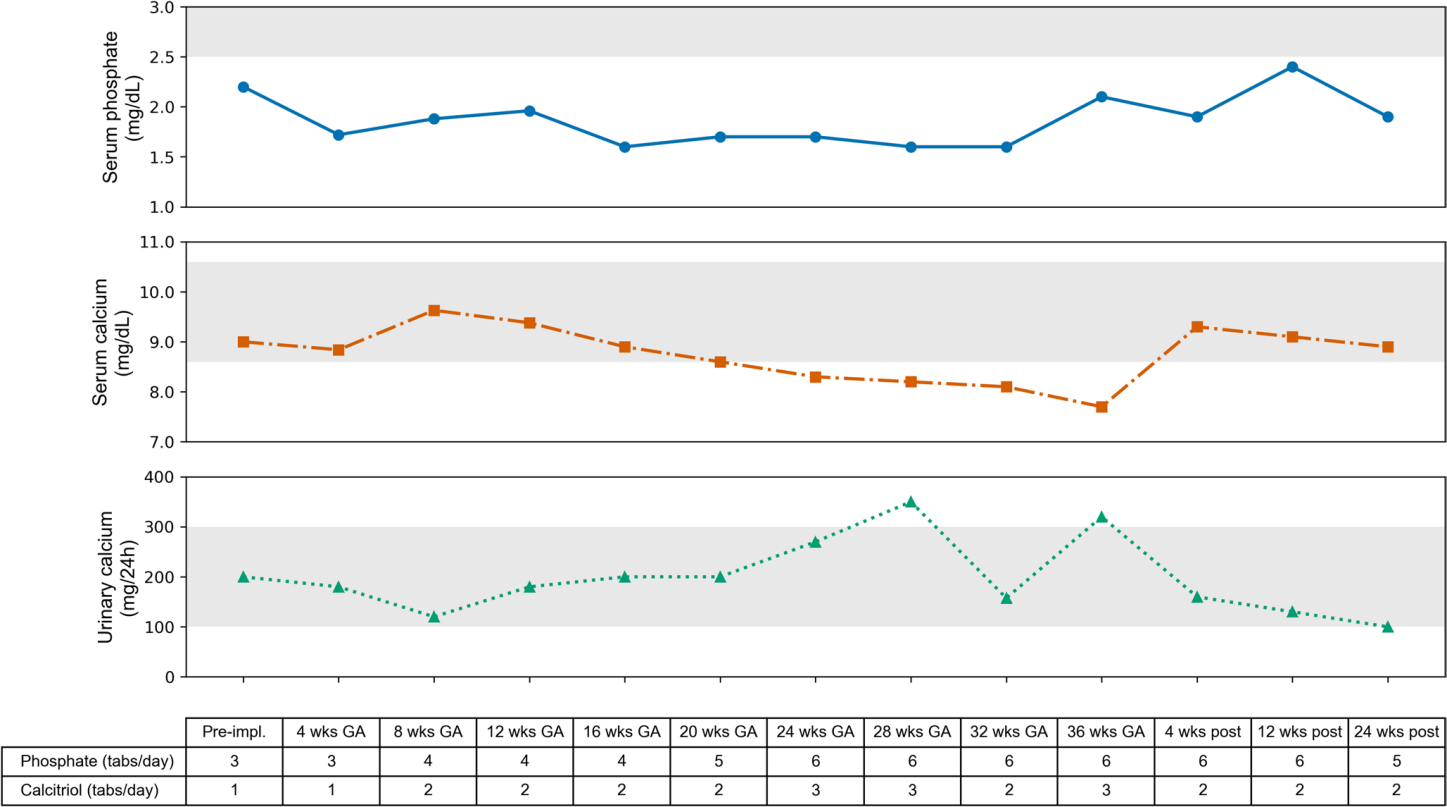
Trend of serum phosphate, serum calcium, and urinary calcium during pregnancy. The shaded grey areas indicate the normal reference ranges for each parameter. The table indicates the number of phosphate (potassium dihydrogen phosphate/disodium hydrogen phosphate, 602 mg/360 mg) and calcitriol (0.25 µg) tablets taken the day before each overnight fasting blood test. *Abbreviations*: GA = gestational age; wks = weeks; Pre-impl. = pre-implantation; post = postpartum

At this point, the patient, who had never been pregnant before, expressed her wish to have a child. After thorough genetic counselling and given the 50% risk of transmitting the PHEX mutation to her offspring, she decided to undergo assisted reproductive technology with preimplantation genetic diagnosis, which allowed the selection and transfer of an embryo not carrying the PHEX mutation. Since the use of burosumab is not recommended during pregnancy, we informed the patient of the need to discontinue this therapy and, if necessary, to resume conventional treatment for XLH, given the previous detection of pseudofractures and the possible increased mechanical load on the lower limbs associated with pregnancy. The patient then discontinued burosumab after a period of 8 months of treatment and in September 2024, the patient underwent embryo implantation, which resulted in a confirmed pregnancy. Concurrently, conventional oral therapy was reintroduced, consisting of phosphate supplements (potassium dihydrogen phosphate/disodium hydrogen phosphate 602 mg/360 mg, three tablets daily) and calcitriol (0.25 µg, once daily), while cholecalciferol supplementation was continued. Clinically, the patient reported a mild increase in muscle weakness and stiffness during pregnancy compared with her condition prior to conception. The patient underwent monthly laboratory monitoring. Trends in serum calcium, serum phosphate, and urinary calcium levels, along with the corresponding therapy at the time of sampling, are presented in Fig. 2. At 36 weeks of gestation, following an attempt to increase calcitriol supplementation, blood tests revealed an increase in urinary calcium excretion (320 mg/24 h) but low calcium levels (7.7 mg/dL). A therapy with calcium carbonate 500 mg/day was then initiated and continued after delivery. Serum creatinine, 25-hydroxyvitamin D (25[OH]D), and PTH levels were also monitored and remained within the reference range throughout gestation. The pregnancy progressed uneventfully, and the patient subsequently delivered via cesarean section at 39 weeks of gestation in June 2025, without intraoperative complications. The newborn was a female, born at term in good health, with appropriate anthropometric measurements for gestational age and no immediate postnatal complications.

At one month postpartum, the patient was receiving phosphate supplementation (potassium dihydrogen phosphate/disodium hydrogen phosphate 602 mg/360 mg, six tablets daily), calcitriol (0.25 µg twice daily), calcium carbonate and cholecalciferol. She reported subjective well-being, good pain control, and laboratory tests revealed serum phosphate of 1.9 mg/dL, serum calcium of 9.3 mg/dL, urinary calcium of 160 mg/24 h, and PTH levels within the normal range. The longest available follow-up was at 24 weeks postpartum, when the patient showed a trend toward increasing serum phosphate levels, although they remained below the normal range, normalization of serum calcium despite discontinuation of oral calcium supplementation, and a reduction in urinary calcium excretion (Fig. 2). Clinically, the patient reported persistent functional impairment, with a WOMAC total score of 42, and a BPI-SF worst pain score of 6.

## Literature Search and Selection

A systematic literature search was performed across MEDLINE/PubMed, EMBASE, and SCOPUS through July 2025 to identify studies reporting on pregnancy in women with hypophosphatemia of any etiology. Relevant keywords were combined with pregnancy-related terms, and additional articles were identified through manual screening of reference lists. Titles, abstracts, and full texts were reviewed independently by two investigators (GPA and MPF). Studies were eligible for inclusion if they were original articles, case reports, case series, or reviews reporting on pregnancy in women with hypophosphatemia of any etiology (including XLH, TIO, and other hypophosphatemic rickets). Studies had to describe maternal management, treatment modifications, pregnancy outcomes, or neonatal outcomes. Exclusion criteria were articles not in English, studies without specific data on pregnancy in hypophosphatemia, and animal studies. Reported cases of patients with hypophosphatemia managed during pregnancy are summarized in Table 2.

**Table 2:** Summary of published cases of hypophosphatemia managed during pregnancy

Authors (year)	Patient Condition	Patient age (years)	Other relevant PMH	Hypophosphatemia-related Treatment Before Pregnancy	Hypophosphatemia-related Treatment During Pregnancy	Other Relevant Treatments	Serum phosphate during pregnancy (mg/dL)	Pregnancy Outcome	Comments
Dobrowolska-Redo A. et al. (2018)	XLH	Not reported	Arterial hypertension, aortic valve insufficiency	Alphacalcidol until 19 years of age	None	Antihypertensive therapy (metoprolol, L-methyldopa)	1.72	Severe symmetric IUGR; cesarean section at 34 weeks; live female infant (1570 g), negative for XLH on genetic testing, good neonatal outcome	The authors primarily attributed the adverse pregnancy outcome to maternal cardiovascular comorbidities rather than to the absence of XLH-specific treatment
Muniz CR et al. (2021)	TIO	32	None	None	None	None	Not reported	Pregnancy not compromised; fetal development normal; delivery uneventful	The authors report that the diagnosis of TIO was made postpartum and that, despite untreated maternal hypophosphatemia, there were no adverse consequences for the newborn
Sum M. et al. (2021)	TIO	28	None	Calcitriol + oral phosphate supplementation	Continuation of phosphate and calcitriol therapy	None	1.60–2.10	Term pregnancy; planned cesarean delivery; healthy female infant; uneventful maternal and neonatal outcome	The authors report that close biochemical monitoring and dose adjustments to maintain normal phosphate levels resulted in the uncomplicated delivery of a full-term healthy infant
Ali DS et al. (2023)*	XLH	30	None	Phosphate and calcitriol since childhood	Continuation of phosphate and calcitriol with upward dose titration (higher requirements in 2nd and 3rd trimester; calcitriol limited by hypercalciuria)	None	2.44–2.48	Induction at 37 weeks due to cholestasis; vaginal delivery; male infant 2.69 kg, genetic testing positive for PHEX mutation	The authors report close biochemical and clinical monitoring every 3 weeks
Truong TBN et al. (2024)	TIO	Not specified (in her 30 s)	None	None	None	None	0.81	Full-term pregnancy; cesarean section; healthy male infant 3200 g; normal neonatal development	The authors report that the diagnosis of TIO was made postpartum and that, despite untreated maternal hypophosphatemia, there were no adverse consequences for the newborn
Dogra PM et al. (2025)	ADHR	33	None	None	Oral phosphate	Analgesics	0.99	Spontaneous abortion at 16 weeks; uneventful post-abortion course	The authors did not attribute the pregnancy loss to the disease or its treatment, but highlighted that in ADHR, iron deficiency anemia should also be considered, as it is a known trigger for clinical manifestations of this condition

*ADHR* Autosomal Dominant Hypophosphatemic Rickets, *IUGR* Intrauterine Growth Restriction, *PMH* Past Medical History, *TIO* Tumor-induced Osteomalacia, *XLH* X-linked hypophosphatemia. *This reference corresponds to a conference abstract, not a full peer-reviewed article

## Discussion and Literature Review

Phosphate homeostasis is critical for both fetal and maternal skeletal mineralization, with approximately 20 g of phosphorus transferred from the maternal circulation to the fetus by the end of gestation, mainly during the third trimester [9]. To meet the increased maternal and fetal mineral requirements, maternal intestinal absorption of calcium and phosphate approximately doubles during pregnancy, a process largely driven by elevated levels of 1,25[OH]_2_D [10]. Despite these profound changes, serum phosphate concentrations in healthy pregnant women typically remain within the normal range, reflecting a finely tuned balance between increased fetal demands and maternal regulatory mechanisms [10]. PTH is normally a key stimulator of 1,25[OH]_2_D synthesis; however, since PTH levels decrease during pregnancy, it is likely that alternative factors—such as PTH-related protein (PTHrP), estradiol, prolactin, and placental lactogen—drive renal 1α-hydroxylase (CYP27B1) activity to sustain 1,25[OH]_2_D production [9]. In XLH models, this adaptation appears to override the expected suppressive effects of elevated FGF23 on 1,25[OH]_2_D synthesis, as maternal 1,25[OH]_2_D levels rise to concentrations comparable to those observed in normal pregnancies despite persistently high circulating FGF23 [11, 12]. Nevertheless, some case reports have documented pregnancies in women with XLH with ongoing hypophosphatemia, yet without adverse maternal or fetal outcomes [10].

Although no evidence-based recommendations are currently available to guide biochemical monitoring during pregnancy, a recent global survey of expert opinion on XLH by Ali et al. suggests that more frequent monitoring may be warranted due to the significant physiological changes in mineral metabolism that occur during this period [7]. In particular, it is advised to measure serum creatinine and calcium once every trimester, and to check fasting serum phosphorus at least once during pregnancy for all women with XLH [7]. In general, in hypophosphatemic disorders, it seems reasonable to maintain serum phosphate levels within or near the normal range by initiating phosphate supplementation and/or calcitriol therapy if not already in use, or by continuing such treatment with possible dose adjustments if already ongoing, in order to optimize fetal mineralization and preserve maternal skeletal health [13]. Calcitriol and phosphate salts are classified as Food and Drug Administration (FDA) pregnancy category C, indicating that potential fetal risks cannot be excluded based on animal studies [7]. However, the potential maternal and fetal benefits of treatment may be greater than these risks [7].

In our literature review, we identified the following cases of patients with hypophosphatemia managed during pregnancy. Dobrowolska-Redo [14] reported a case of XLH in which phosphate and calcitriol were not administered during pregnancy, likely due to the risk of treatment-related adverse effects (e.g., nephrocalcinosis, hyperparathyroidism) in a patient with pre-existing arterial hypertension and aortic valve insufficiency. The pregnancy was complicated by severe symmetric intrauterine growth restriction (IUGR), which the authors attributed primarily to maternal cardiovascular comorbidities rather than to the absence of XLH-specific treatment. Ali [15] described the case of a woman with XLH who continued phosphate and calcitriol therapy throughout pregnancy, with dosage adjustments based on biochemical monitoring. The pregnancy progressed uneventfully, and a healthy male infant carrying the same PHEX mutation was delivered at term. Dogra [16] presented the case of a woman with autosomal dominant hypophosphatemic rickets (ADHR) whose pregnancy ended in spontaneous abortion at 16 weeks. The patient was receiving phosphate and vitamin D supplementation at the time. She had previously had a successful pregnancy three years earlier. The authors did not attribute the pregnancy loss to the disease or its treatment, but highlighted that in ADHR, iron deficiency anemia should also be considered, as it is a known trigger for clinical manifestations of this condition—unlike other forms of hypophosphatemic rickets. Truong [17] and Muniz [18] reported two cases of women in their 30 s with severe hypophosphatemia due to undiagnosed TIO, who did not receive conventional treatment during pregnancy. Both delivered healthy infants with normal growth and development but experienced significant bone pain, likely attributable to the underlying disease. On the contrary, Sum et al. [19] reported the case of a 28-year-old who was diagnosed with TIO prior to pregnancy and continued calcitriol and phosphate therapy throughout gestation, allowing for biochemical monitoring and dose adjustments to maintain normal phosphate levels, ultimately resulting in the uncomplicated delivery of a full-term healthy infant. During pregnancy, the patient reported severe joint pain causing reduced mobility and requiring family support. After delivery, imaging identified the tumor causing TIO, which was surgically removed. FGF23 levels normalized, and symptoms improved significantly.

Given the scarce evidence available in the literature, we opted to resume phosphate and calcitriol therapy during pregnancy in our patient, due to the presence of multiple pseudofractures prior to conception, reflecting severe skeletal fragility, and the fact that such fractures had occurred in the past despite ongoing conventional treatment. Moreover, recent reports have raised concerns regarding the worsening of symptoms and serum phosphate levels following burosumab withdrawal [20]. In addition, cholecalciferol supplementation was maintained throughout pregnancy, in line with current recommendations for healthy pregnant women, to support both maternal and offspring health [21]. We also opted for more frequent monitoring than currently recommended [7], to mitigate the risk of side effects such as hypercalcemia or hypercalciuria, which may be increased during pregnancy due to physiological elevations in 1,25[OH]_2_D levels. This approach allowed for close biochemical surveillance and facilitated the tailoring of therapy, with the aim of maintaining serum phosphate within or near the lower limit of the normal range, while avoiding excessive fluctuations in serum and urinary calcium, and keeping serum creatinine and PTH levels within normal limits. As expected, an increased demand for serum phosphate was observed, leading to a gradual escalation of phosphate supplementation to meet the elevated fetal phosphorus requirements of the third trimester, whereas the calcitriol dose was reduced during the same period due to hypercalciuria, likely attributable to increased endogenous 1,25[OH]_2_D production. In the third trimester, low total serum calcium was also observed, prompting empiric initiation of oral calcium supplementation; unfortunately, neither serum albumin nor ionized calcium levels were available, and it cannot be excluded that this finding was due to a pregnancy-related hemodilution effect. However, it should be noted that, despite the strategy of frequent monitoring and therapy being adjusted accordingly, achieving an adequate control of mineral metabolism with conventional therapy in this patient remained challenging and not fully optimal, and in similar situations the possibility of complications—such as renal manifestations related to hypercalciuria—cannot be excluded.

Regarding the mode of delivery, the available literature indicates that data in women with XLH are heterogeneous, with reported cesarean section rates ranging from approximately 14%, similar to the general population, to as high as 76% [7]. The reasons for cesarean delivery were varied, but most often the decision reflected the preference of both obstetricians and affected women to opt for elective cesarean section due to the potential complications of labor associated with short maternal stature, concerns about a narrowed birth canal, and the consequent risk of obstructed labor [22]. In addition, a theoretical risk of pelvic injury in women with impaired bone mineralization due to osteomalacia should also be considered. Given the patient’s biochemical course and the history of previous pseudofractures despite conventional treatment, a cesarean section was chosen in our case; however, it should be emphasized that specific recommendations on this aspect are currently lacking.

Regarding the breastfeeding period, conventional therapy was continued, with gradual biochemical improvement allowing a partial dose reduction. After cessation of breastfeeding, if clinical symptoms persist, high-dose conventional therapy continues to be required, or if there is evidence of incomplete healing of pseudofractures, in our opinion resumption of burosumab therapy would be a reasonable and clinically appropriate option.

To the best of our knowledge, this is the first documented case in the literature detailing the clinical course and management of a pregnancy in which therapy was transitioned from burosumab to conventional treatment, thereby providing novel insights into therapeutic decision-making in this rare and challenging scenario, although further studies are warranted.

In conclusion, to date, there is no robust evidence to guide the management of hypophosphatemia during pregnancy, and clinical decisions must rely on pathophysiological reasoning and individual patient characteristics. Nevertheless, in women with severe hypophosphatemia or marked skeletal involvement, the initiation or continuation of conventional treatment with phosphate and active vitamin D analogues during pregnancy appears to be a reasonable approach to prevent maternal complications, such as pseudofractures and debilitating bone pain, as well as to support optimal fetal skeletal development. Close biochemical monitoring is strongly recommended to minimize the risk of adverse effects, which may be exacerbated by pregnancy-related changes in mineral metabolism. Further studies are needed to better characterize the course of XLH and other hypophosphatemic disorders during pregnancy and to develop evidence-based recommendations for their management.

## Data Availability

The data that has been used is confidential.
